# Supplementary material for: Chromatin-informed inference of transcriptional programs in gynecologic and basal breast cancers
Source: Nat Commun. 2019 Sep 25;10:4369. doi: 10.1038/s41467-019-12291-6 (PMC6761109; doi:10.1038/s41467-019-12291-6)
Supplement: Supplementary file 1 — Supplementary Information [file 41467_2019_12291_MOESM1_ESM.pdf]

Supplementary Information for:

Osmanbeyoglu et al. "Chromatin-informed inference of transcriptional programs in gynecologic and basal breast cancers"

Table of Contents

1: Supplementary Figures
2: Supplementary Tables

Supplementary Figures

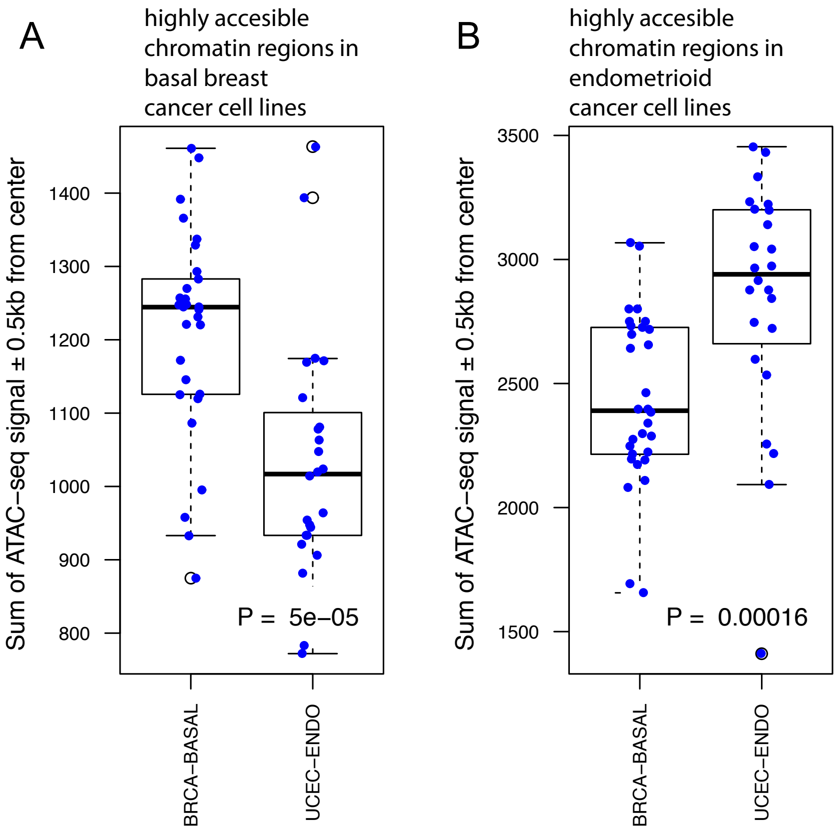

Supplementary Figure 1. Comparison of ATAC-seq profiles of BRCA-BASAL and UCEC-ENDO tumors in differential chromatin accessibility regions from basal breast and endometrial cell lines. **(A)** BRCA-BASAL tumors have significantly higher sum of ATAC-seq signal compared to UCEC-ENDO tumors at loci with increased chromatin accessibility in basal breast cancer cell lines compared to endometrial cell lines ($P < 10^{-4}$, one-sided Wilcoxon signed-rank test); **(B)** UCEC-ENDO tumors have significantly higher sum of ATAC-seq signal compared to BRCA-BASAL tumors at loci with increased chromatin accessibility in endometrial cell lines compared to basal breast cancer cell lines ($P = 0.00016$, one-sided Wilcoxon signed-rank test). Box edges represent the upper and lower quantile with median value shown as bold line in the middle of the box. Whiskers represent 1.5 times the quantile of the data. Source data are provided as a Source Data file.

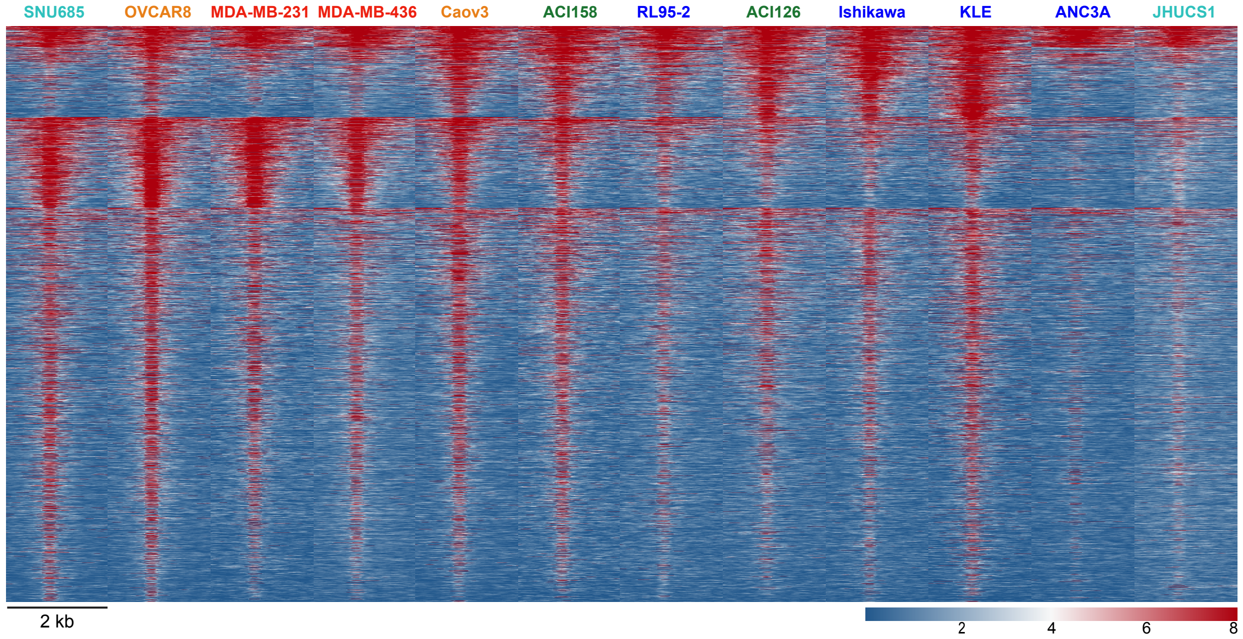

Supplementary Figure 2. Chromatin accessibility heat map grouped by differential accessibility patterns. Each row represents one of 40,000 selected peaks (differentially accessible between at least one histologic type comparison; FDR < 0.0001, log2(fold change; FC) > 3).

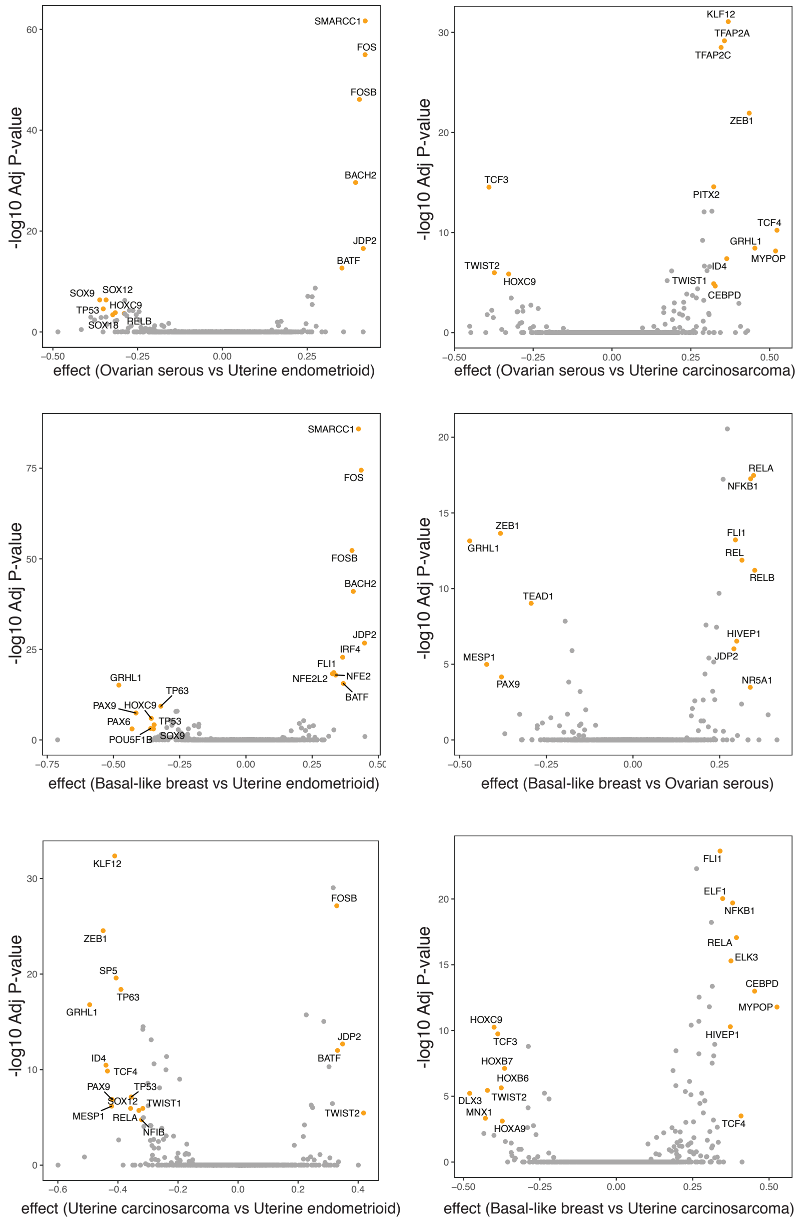

Supplementary Figure 3. Pairwise comparison of transcription factor motifs enriched in differentially accessible regions in cell lines. Volcano plot showing effect size versus –log10(adjusted *P*), using a Bonferroni correction to adjust *P* values for each plot. TF symbol annotations are written where the absolute value of the effect size is in at least top 30 and adjusted $P < 10^{-3}$. The foreground occurrence is the number of peaks containing a particular TF motif within the group of 5000 upregulated or 5000 downregulated peaks according to log2 fold change read counts, respectively. The background occurrence is the number of peaks containing a particular TF motif found among all the differentially accessible peaks (ovarian serous cell lines: OVCAR8, Caov3; basal-like breast: MDA-MB-231, MDA-MB-436; uterine serous cell lines: ACI158, ACI126; uterine carcinosarcoma: JHUCS1, SNU685; uterine endometrioid: Ishikawa, KLE, RL95-1, AN3CA).

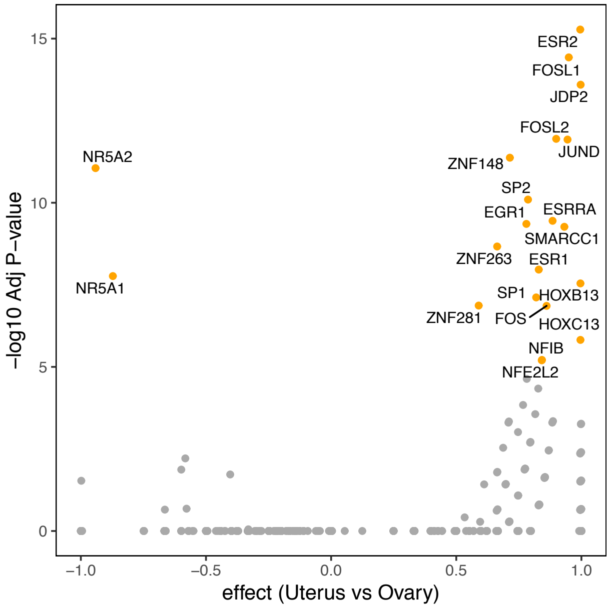

Supplementary Figure 4: Pairwise comparison of transcription factor motifs enriched in differentially accessible regions in normal tissues Uterus vs Ovary. Volcano plot showing effect size versus –log10(*P*), using a Bonferroni correction to adjust *P* values for each plot. TF symbol annotations are written where the adjusted $P < 10^5$. The foreground occurrence is the number of peaks containing a particular TF motif within the group of differential accessible peaks according to log2 fold-change read counts, respectively. The background occurrence is the number of peaks containing a particular TF motif found among all the differentially accessible peaks.

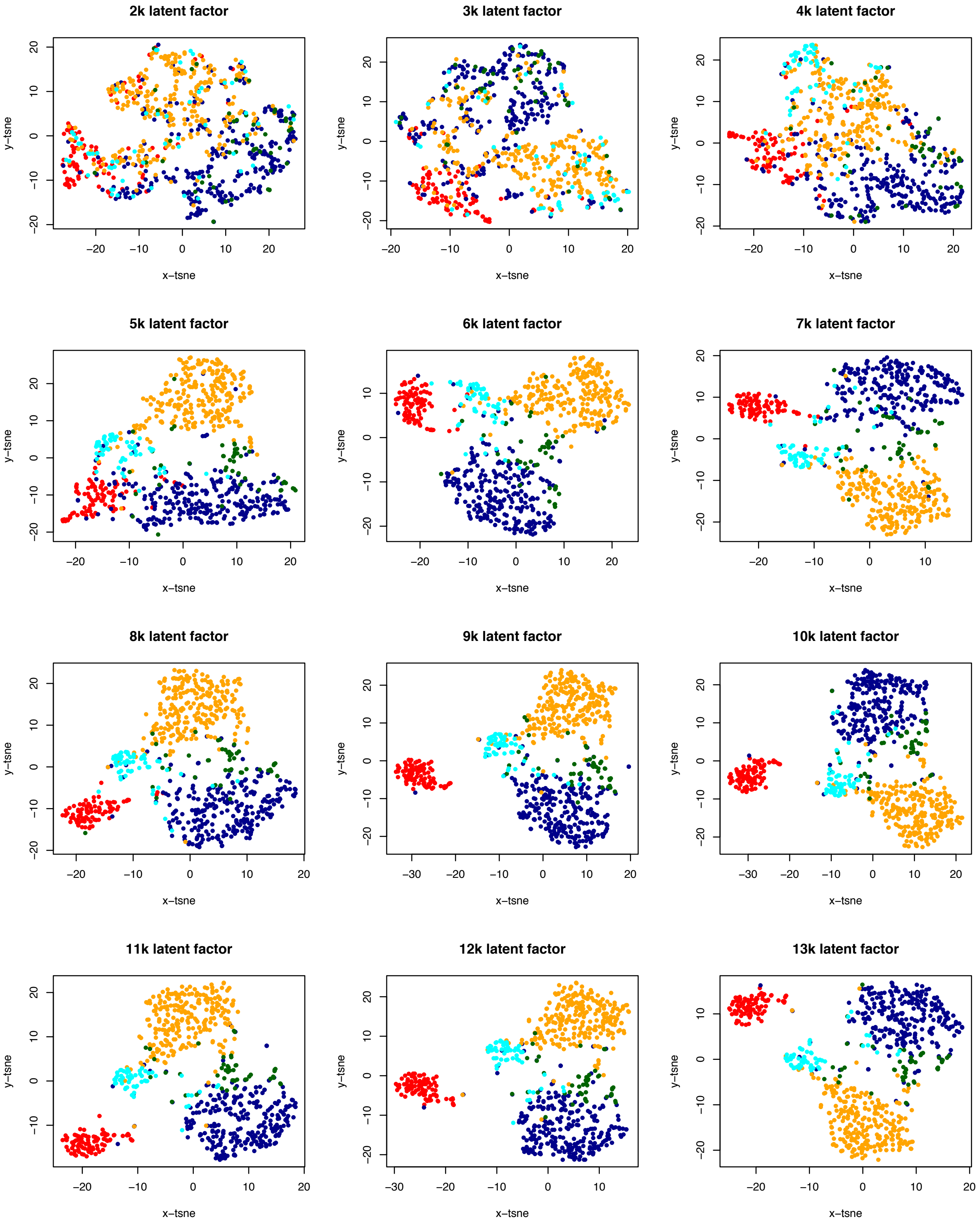

Supplementary Figure 5. t-SNE projections of inferred TF activities with varying K values. 10-fold cross validation results, reported as mean and standard deviation Spearman correlation values, are: K = 2: 0.383±0.019; K = 3: 0.383±0.016; K = 4: 0.384±0.016; K = 5: 0.384±0.016; K = 6: 0.384±0.016; K = 7: 0.384±0.016; K = 8: 0.384±0.016; K = 9: 0.384±0.016.

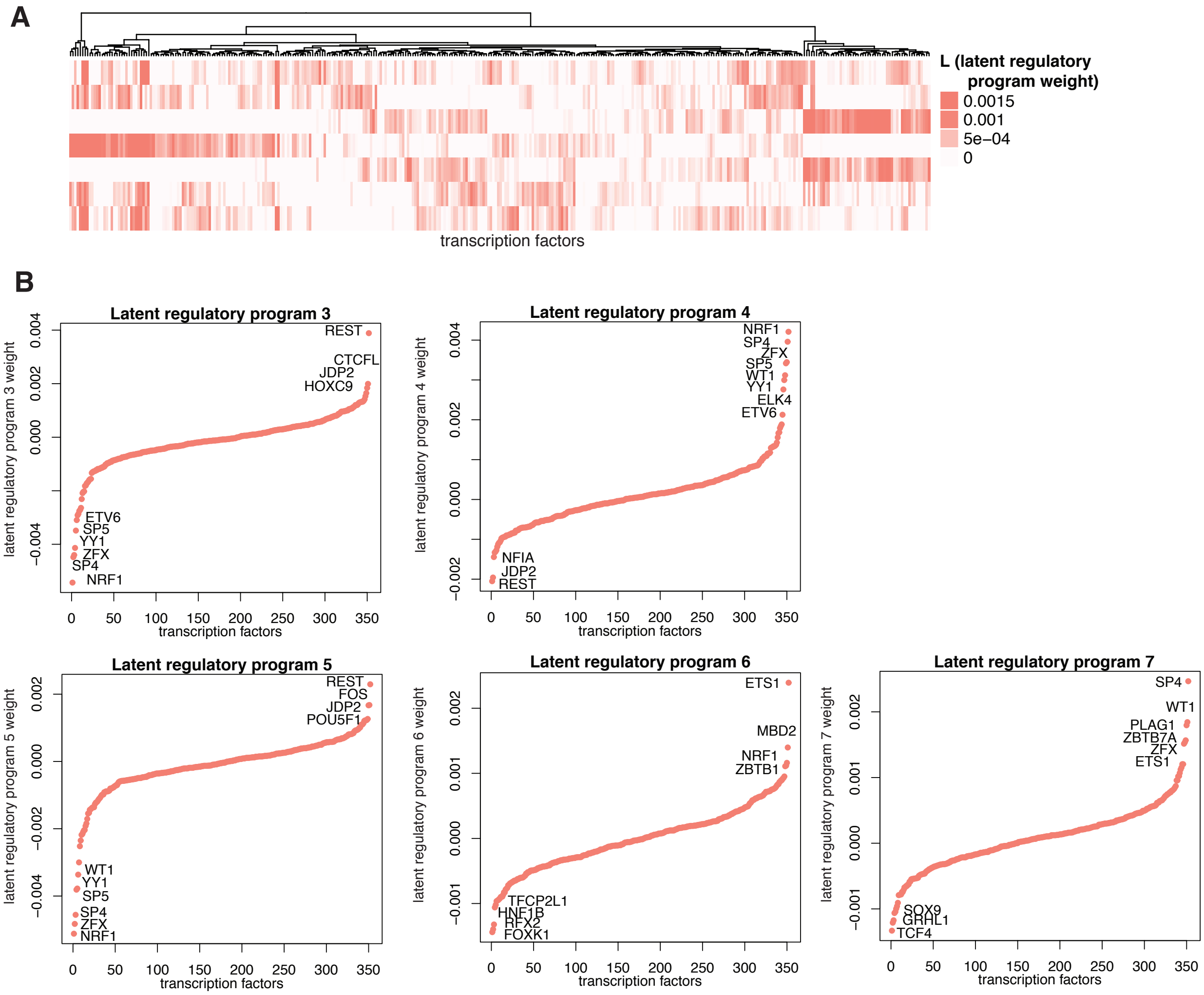

Supplementary Figure 6. (A) Heatmap of latent regulatory programs/tasks (**L**). **(B)** Plots show latent regulatory program weights. Regulators are ranked based on increase in magnitude of coefficients. Source data are provided as a Source Data File.

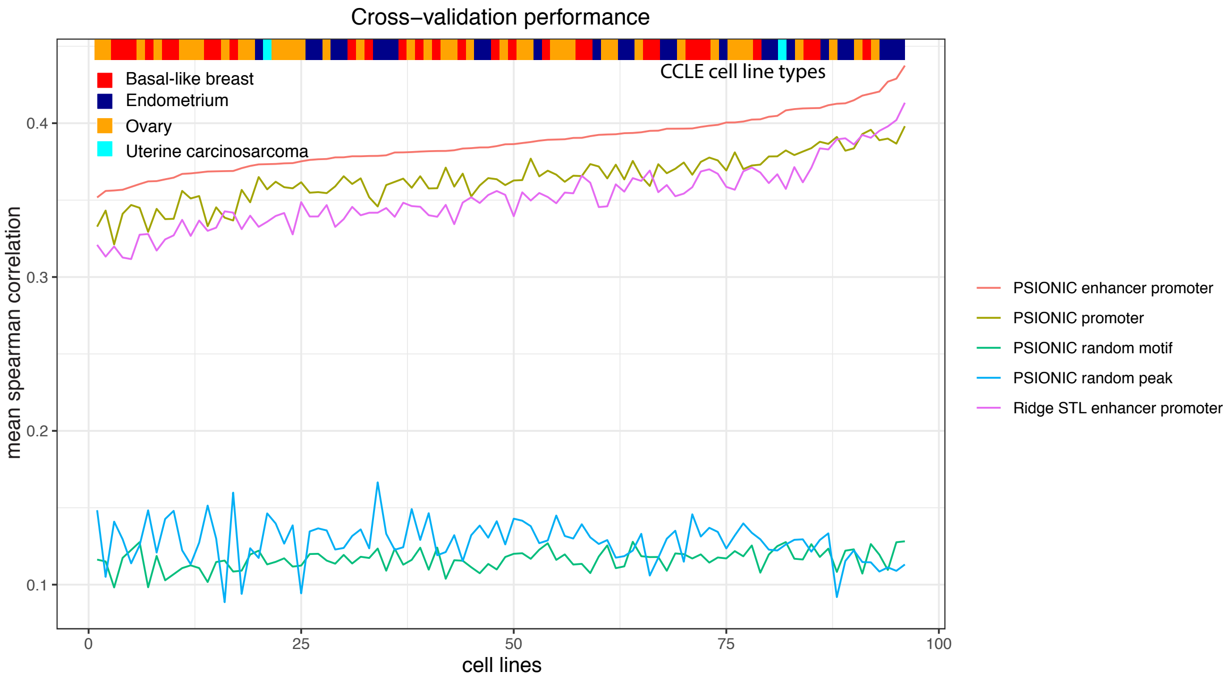

Supplementary Figure 7. PSIONIC and STL regression models predict differential expression of held-out genes and subtypes of cell lines from CCLE. Plot showing Spearman correlations between predicted and actual gene expression changes for all samples, sorted based on performance of the PSIONIC model using enhancer and promoter TF binding sites. For each method and each cell line, the Spearman correlation is computed using 10-fold cross-validation on held-out genes. Using TF binding sites from enhancer promoter as features (mean ρ = 0.387± 0.018) is significantly better than when we randomized motif hits for each chromatin accessible region across all motifs (mean ρ = 0.116± 0.007; $P < 10^{-32}$, one-sided Wilcoxon signed-rank test), or if we randomized accessible regions for each motif, then assigned to the nearest gene (mean ρ = 0.128± 0.014; $P < 10^{-32}$, one-sided Wilcoxon signed-rank test). PSIONIC models with motif data from promoter and enhancer regions outperformed models where only motif hits in promoter regions were used (mean ρ = 0.364± 0.016; $P < 10^{-16}$, one-sided Wilcoxon signed-rank test) and STL approach based on ridge regression (mean ρ = 0.352± 021; $P < 10^{-21}$, one-sided Wilcoxon signed-rank test). TCGA tumor types are shown in the top bar.

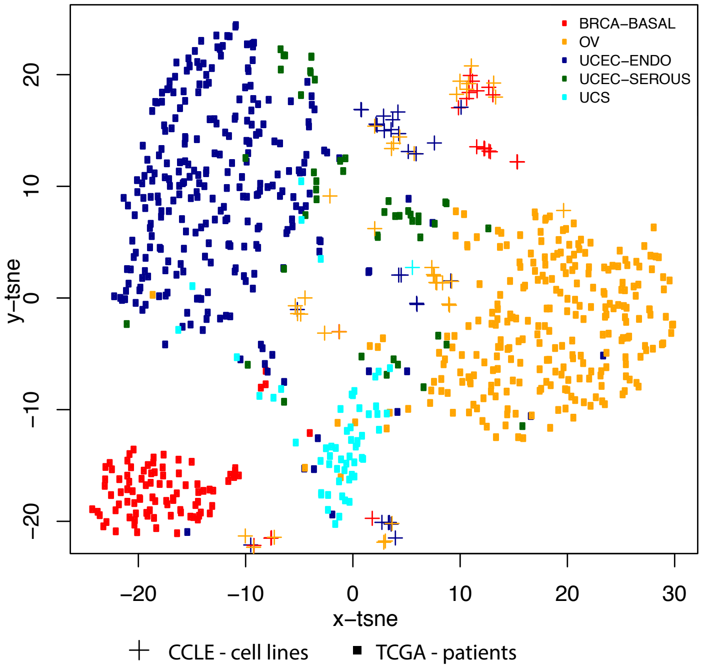

Supplementary Figure 8. t-SNE projections of mean centered inferred TF activities for TCGA patients (as denoted with squares) and CCLE cell lines (as denoted with plus sign). Source data are provided as a Source Data File.

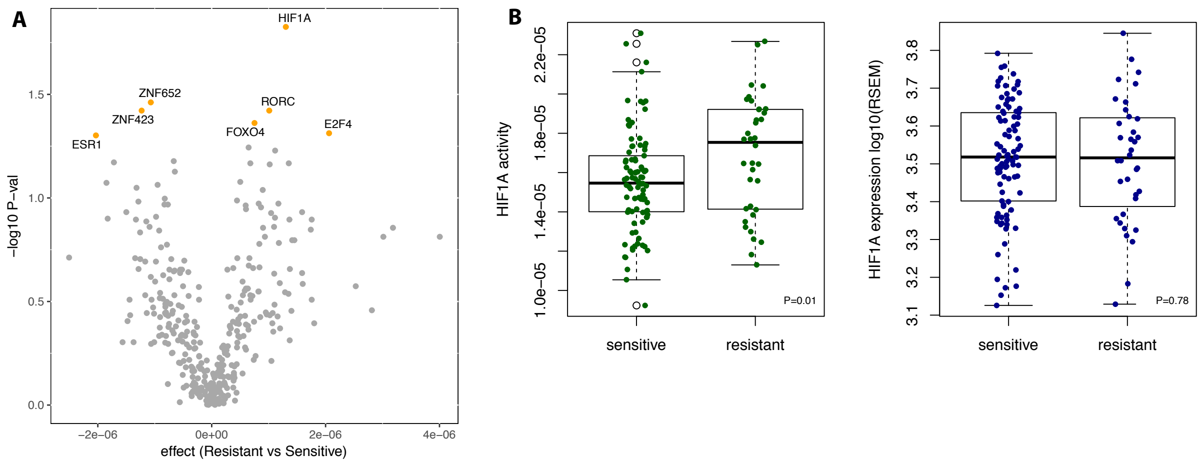

Supplementary Figure 9. (A) The mean inferred TF activity difference in platinum resistant and sensitive patients is plotted on the *x* axis, and *P* values from *t*-test is plotted on the *y* axis (–log10 scale) for OV cohort. TFs associated with response ($P < 0.02$, t-test) are colored in orange. **(B)** HIF1A differential inferred TF activities in platinum resistant and sensitive patients tumors ($P < 10^{-1}$, Wilcoxon rank-sum test). Importantly, HIF1A gene expression is not statistically significantly different in gene expression level (right side). Box edges represent the upper and lower quantile with median value shown as bold line in the middle of the box. Whiskers represent 1.5 times the quantile of the data.

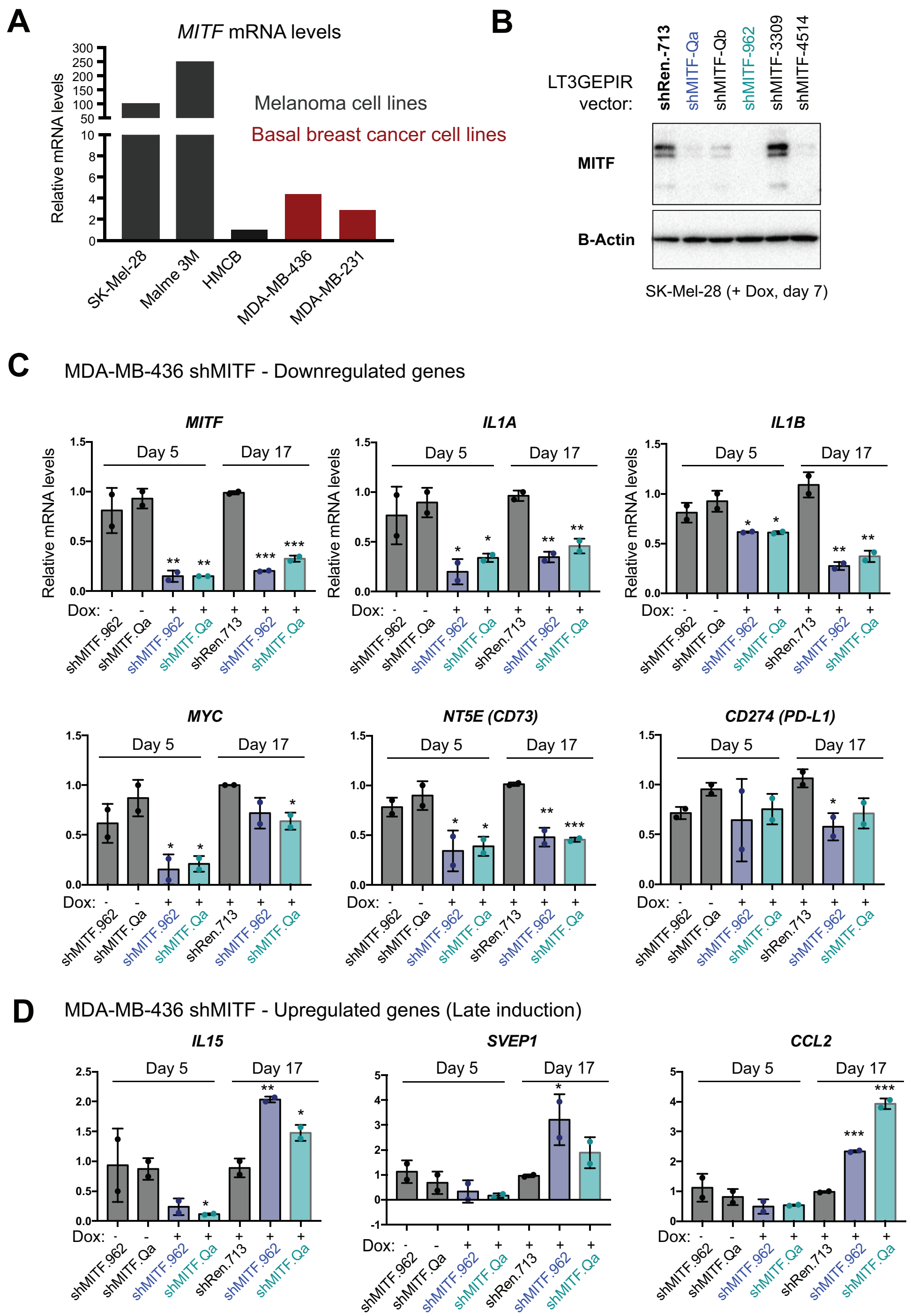

Supplementary Figure 10. Validation shRNA-mediated transcriptional changes following MITF knockdown. (A) MITF expression in basal breast cancer cells versus melanoma cell lines, showing basal expression in these basal breast cancer cells comparable to MITF-lowly expressed melanoma line HMCB. **(B)** Validation of shRNA-mediated MITF knockdown efficiency by the indicated inducible shRNA vector. **(C, D)** RT-qPCR validation of RNA-seq results showing target gene modulation at day 5 and 17. Data are presented as means ± SD of two biological replicates. "*" stands for (unpaired one-tailed Student's T-test) $P < 0.05$, "**" for $P < 0.01$, and "***" for $P < 0.001$. Source data are provided as a Source Data File.

Supplementary Tables

Supplementary Table 1. Summary of cell line ATAC-seq data.

Cell line	Tumor type	Reproducible peaks	Atlas peaks
MDA-MB-231	Basal breast	69,136	102,981
MDA-MB-436		77,999	
CAOV3	Ovarian adenocarcinoma	102,048	153,681
OVCAR8		110,600	
JHUCS	Uterine carcinosarcoma	50,483	135,948
SNU685		121,132	
AN3CA	Uterine endometrioid adenocarcinoma	54,668	147,024
Ishikawa		68,207	
KLE		83,824	
RL95_2		50,221	
ACI-126	Uterine serous adenocarcinoma	56,175	93,976
ACI-158		69,918	

Supplementary Table 2. Survival analysis for BRCA-BASAL. Cox proportional hazards regression models for TF regulators based on inferred TF activity. We added clinical stage and age as background factors.

Covariate (TF)	P-value	FDR	Coeff
MITF	7.7E-05	0.011	36.1
SOX4	9.1E-05	0.011	29.9
HOXB9	9.8E-05	0.011	27.0
PITX1	1.5E-04	0.013	-22.5
ZNF282	1.9E-04	0.014	-63.3
ZEB1	2.4E-04	0.014	-42.9
NR5A1	3.3E-04	0.014	31.4
SOX8	3.5E-04	0.014	22.0
ATF5	3.8E-04	0.014	15.8
HOXB13	4.2E-04	0.014	74.7
FOXP3	4.8E-04	0.014	25.1
FOXM1	4.9E-04	0.014	33.6
NFIA	5.5E-04	0.015	21.4
HOXA3	6.1E-04	0.015	15.7
MXI1	6.5E-04	0.015	-26.4
KLF12	7.0E-04	0.015	-11.6
TCF7L2	8.9E-04	0.018	20.1
BARX1	9.0E-04	0.018	-51.1

Supplementary Table 3. Survival analysis for UCEC-SEROUS. Cox proportional hazards regression models for TF regulators based on inferred TF activity. We added clinical stage and age as background factors.

Covariate (TF)	P-value	FDR	Coeff
TFEC	5.1E-08	$< 10^{-3}$	-22.8
ETV6	3.3E-06	0.001	-9.9
MLL	5.7E-06	0.001	-16.9
CREB1	7.0E-06	0.001	-21.4
POU5F1	8.0E-06	0.001	18.2
NFYB	2.0E-05	0.001	-26.0
STAT6	2.3E-05	0.001	18.9
SP5	4.8E-05	0.002	-4.9
FOXA2	5.2E-05	0.002	21.9
ZNF143	6.6E-05	0.002	-10.8
MAX	8.4E-05	0.003	14.9
TCF3	1.1E-04	0.003	17.3
ELF4	1.5E-04	0.004	-12.2
PAX6	1.9E-04	0.005	21.1
ZNF589	2.1E-04	0.005	29.7
RXRG	2.3E-04	0.005	-16.7
ETS2	2.3E-04	0.005	-24.5
MXI1	2.7E-04	0.005	-21.7
PAX2	2.8E-04	0.005	27.8
TEAD3	3.0E-04	0.005	19.4
GLI2	3.1E-04	0.005	18.9
ZNF76	3.5E-04	0.006	-37.3
PPARD	5.8E-04	0.009	-25.6
IRF2	7.8E-04	0.011	-15.7
CREB3L1	8.0E-04	0.011	-22.5
FOXP3	9.4E-04	0.013	15.1
ZNF32	1.0E-03	0.014	14.2
HOXC8	1.2E-03	0.015	24.9
ATF5	1.6E-03	0.019	12.0

Supplementary Table 4. Survival analysis for UCEC-ENDO. Cox proportional hazards regression models for TF regulators based on inferred TF activity. We added clinical stage and age as background factors.

Covariate (TF)	P-value	FDR	Coeff
MNX1	2.1E-07	0.0001	15.7
ATF6	3.9E-07	0.0001	-13.1
HSF2	5.9E-07	0.0001	22.5
SOX7	1.8E-06	0.0001	-16.2
PGR	2.1E-06	0.0001	28.8
ZNF652	7.2E-06	0.0004	-11.5
RORC	1.2E-05	0.0006	11.3
ZNF784	1.4E-05	0.0006	-20.8
ZBTB4	2.6E-05	0.0010	-17.7
BACH2	5.0E-05	0.0017	17.9
TGIF2	2.1E-04	0.0067	9.3
ZNF143	5.6E-04	0.0165	-7.5

Supplementary Table 5. List of 161 differentially expressed genes identified by RNA-Seq (FDR-adjusted *P* < 0.05 and fold change > 2).

Gene Symbol	baseMean	log2 FoldChange	lfcSE	stat	P-value	padj
AATK	57.50	1.08	0.31	3.51	4.5E-04	5.6E-03
ABCG1	74.33	2.22	0.31	7.21	5.7E-13	6.5E-11
ADTRP	62.20	-1.31	0.26	-5.09	3.6E-07	1.4E-05
AIM1	174.18	1.06	0.19	5.67	1.4E-08	7.8E-07
ALOX5	133.43	1.08	0.27	4.07	4.7E-05	9.2E-04
ALX4	196.15	1.44	0.18	8.23	1.9E-16	3.5E-14
ANGPTL4	280.21	-1.07	0.34	-3.19	1.4E-03	1.4E-02
AOX1	342.48	-1.42	0.13	-10.64	2.0E-26	9.3E-24
APOL1	148.48	1.13	0.24	4.78	1.8E-06	5.7E-05
C10orf114	64.86	1.11	0.30	3.66	2.5E-04	3.6E-03
C5orf4	31.09	1.16	0.42	2.78	5.5E-03	3.9E-02
CA11	100.78	1.22	0.24	5.07	4.1E-07	1.6E-05
CA9	83.40	1.33	0.33	4.03	5.5E-05	1.0E-03
CACNA2D3	122.15	2.03	0.26	7.96	1.7E-15	2.8E-13
CACNG4	74.57	1.41	0.36	3.93	8.4E-05	1.5E-03
CCL2	96.62	1.16	0.31	3.74	1.8E-04	2.8E-03
CCL3	103.70	-1.56	0.23	-6.93	4.3E-12	4.3E-10
CD274	422.63	-1.05	0.29	-3.65	2.7E-04	3.7E-03
CD68	1868.51	-1.08	0.09	-11.42	3.3E-30	1.9E-27
CDC42BPG	96.46	1.28	0.24	5.25	1.6E-07	7.0E-06
CFI	555.01	1.79	0.12	15.54	1.7E-54	3.4E-51
CHI3L1	61.88	1.21	0.32	3.80	1.4E-04	2.3E-03
CHST6	34.01	1.04	0.38	2.73	6.4E-03	4.3E-02
CLUL1	27.74	1.24	0.43	2.88	3.9E-03	3.0E-02
CMPK2	887.39	1.00	0.13	7.94	2.1E-15	3.3E-13
CNTNAP3	126.14	1.27	0.21	6.04	1.5E-09	1.1E-07
COL14A1	46.84	1.10	0.34	3.25	1.2E-03	1.2E-02
COL1A1	387.07	1.11	0.14	7.89	3.2E-15	5.1E-13
CPA4	80.75	1.25	0.35	3.62	2.9E-04	4.0E-03
CTSS	619.72	-1.00	0.14	-7.07	1.5E-12	1.7E-10
CXCL3	319.94	-1.23	0.18	-6.89	5.5E-12	5.4E-10
CYP1B1	1556.57	1.49	0.08	19.76	7.2E-87	3.3E-83
EGR4	38.59	-1.35	0.34	-4.01	6.2E-05	1.2E-03
EMID2	28.22	1.66	0.46	3.62	2.9E-04	4.0E-03
EMX2	115.97	-1.01	0.19	-5.26	1.5E-07	6.7E-06
ENTPD3	17.82	-1.93	0.48	-4.03	5.7E-05	1.1E-03
EPHA7	29.24	1.16	0.42	2.78	5.4E-03	3.9E-02
ESM1	308.16	-3.12	0.17	-18.97	3.0E-80	1.0E-76
FAAH2	54.26	1.24	0.33	3.75	1.8E-04	2.7E-03
FBXO2	32.90	1.21	0.41	2.95	3.2E-03	2.5E-02
FGF1	28.14	-1.44	0.40	-3.55	3.8E-04	4.9E-03
FGF5	146.65	-2.11	0.18	-11.59	4.6E-31	2.9E-28
FMO5	62.05	1.24	0.30	4.17	3.1E-05	6.4E-04
FST	147.27	-1.17	0.18	-6.67	2.7E-11	2.4E-09
GAL	737.99	-1.10	0.11	-10.39	2.8E-25	1.3E-22
GBP4	19.44	1.65	0.54	3.04	2.4E-03	2.0E-02
GNAT1	51.22	1.74	0.34	5.08	3.7E-07	1.5E-05
GPR1	28.90	1.20	0.43	2.77	5.6E-03	3.9E-02
GPR162	245.51	1.24	0.15	8.13	4.4E-16	7.7E-14
GPR20	36.92	1.56	0.39	4.03	5.6E-05	1.1E-03
GPR56	512.99	1.00	0.14	7.44	1.0E-13	1.3E-11
GREM1	2279.16	-1.72	0.09	-19.76	7.2E-87	3.3E-83
GUCY1A2	28.18	1.27	0.43	2.95	3.2E-03	2.5E-02

HCN4	47.45	1.16	0.33	3.57	3.6E-04	4.7E-03
HMGA2	450.99	-1.02	0.11	-9.11	8.4E-20	2.3E-17
HR	53.25	1.33	0.36	3.71	2.0E-04	3.0E-03
IFI44L	274.47	1.32	0.16	8.22	2.0E-16	3.7E-14
IGFBP5	37.99	2.02	0.43	4.74	2.2E-06	6.7E-05
IGFN1	13657.50	-1.21	0.07	-18.28	1.3E-74	2.9E-71
IL11	90.66	-1.16	0.24	-4.91	9.0E-07	3.1E-05
IL12A	35.04	-1.83	0.36	-5.04	4.7E-07	1.8E-05
IL13RA2	110.32	-1.41	0.27	-5.21	1.9E-07	8.2E-06
IL1A	4311.50	-1.40	0.24	-5.74	9.3E-09	5.4E-07
IL1B	9667.75	-1.91	0.08	-22.65	1.3E-113	1.8E-109
IL24	41.97	-2.13	0.40	-5.33	1.0E-07	4.7E-06
ITGB4	825.85	1.22	0.10	12.12	8.5E-34	6.1E-31
ITGB8	137.53	-1.00	0.19	-5.41	6.3E-08	3.1E-06
KANK4	54.24	2.30	0.36	6.39	1.6E-10	1.3E-08
KCNB1	41.19	1.29	0.35	3.63	2.9E-04	3.9E-03
KRT17	289.02	1.29	0.15	8.48	2.2E-17	4.5E-15
LCP1	45.11	-1.08	0.32	-3.39	7.0E-04	7.8E-03
LIF	7149.07	-1.10	0.28	-3.90	9.7E-05	1.7E-03
LINC00324	99.90	1.06	0.27	3.87	1.1E-04	1.8E-03
LOC100505865	29.07	1.38	0.46	3.00	2.7E-03	2.2E-02
LOC645638	74.21	1.39	0.31	4.47	7.7E-06	2.0E-04
LOC646329	74.49	-1.22	0.24	-5.05	4.4E-07	1.7E-05
LRRC33	56.58	-1.26	0.27	-4.69	2.8E-06	8.1E-05
LRRC38	21.45	1.35	0.49	2.77	5.7E-03	4.0E-02
MAL	27.35	1.34	0.47	2.85	4.4E-03	3.3E-02
MAP2	100.26	1.81	0.27	6.80	1.0E-11	9.6E-10
43528	512.89	-1.42	0.10	-13.97	2.5E-44	2.6E-41
MEST	33.15	2.23	0.48	4.62	3.9E-06	1.1E-04
MIR210HG	61.24	1.47	0.34	4.28	1.9E-05	4.1E-04
MITF	353.46	-1.99	0.40	-5.05	4.4E-07	1.7E-05
MLXIPL	77.68	1.41	0.29	4.93	8.2E-07	2.9E-05
MME	212.78	-1.11	0.15	-7.34	2.1E-13	2.6E-11
MMP1	287.92	-1.30	0.42	-3.08	2.1E-03	1.9E-02
MPP4	36.69	-1.13	0.35	-3.26	1.1E-03	1.1E-02
MYO1G	25.04	1.54	0.48	3.21	1.3E-03	1.3E-02
MYPN	346.38	-1.46	0.15	-9.98	1.9E-23	8.0E-21
NAV3	820.45	-1.20	0.11	-11.28	1.7E-29	9.3E-27
NIPAL1	91.47	-1.04	0.22	-4.73	2.3E-06	6.9E-05
NOXA1	127.05	1.10	0.21	5.26	1.4E-07	6.5E-06
NRTN	33.35	1.05	0.39	2.69	7.2E-03	4.7E-02
NT5E	1614.33	-1.06	0.09	-11.82	2.9E-32	1.9E-29
OAS1	41.50	1.81	0.38	4.77	1.9E-06	5.9E-05
OLFML2A	928.27	1.34	0.35	3.82	1.4E-04	2.2E-03
PADI2	17.47	1.47	0.55	2.69	7.2E-03	4.7E-02
PAPL	103.07	-1.25	0.20	-6.18	6.5E-10	5.0E-08
PCDH1	69.85	1.15	0.29	3.94	8.0E-05	1.4E-03
PCDH7	72.61	1.05	0.27	3.82	1.3E-04	2.2E-03
PGM5	36.98	-1.67	0.35	-4.79	1.7E-06	5.4E-05
PHACTR1	150.91	-1.15	0.19	-6.11	1.0E-09	7.5E-08
PIK3C2B	387.83	1.02	0.13	7.98	1.4E-15	2.4E-13
PKIB	105.07	-1.17	0.20	-5.88	4.1E-09	2.6E-07
PKNOX2	17.37	2.59	0.68	3.83	1.3E-04	2.1E-03
PLCL1	35.44	1.01	0.37	2.71	6.7E-03	4.5E-02
PLXNA4	48.06	1.09	0.33	3.28	1.0E-03	1.1E-02
PPL	163.35	1.63	0.43	3.80	1.5E-04	2.3E-03
PRR15	49.17	1.03	0.31	3.30	9.7E-04	1.0E-02
RARRES3	167.33	1.60	0.23	6.94	4.0E-12	4.1E-10
RPLP0P2	31.42	-1.15	0.37	-3.09	2.0E-03	1.8E-02

RSAD2	2971.92	1.55	0.31	4.97	6.6E-07	2.4E-05
RSPO3	87.35	-1.26	0.22	-5.69	1.3E-08	7.1E-07
RXRG	205.51	1.43	0.19	7.48	7.7E-14	1.0E-11
S100A4	120.67	1.62	0.23	7.21	5.6E-13	6.4E-11
S100A8	69.62	4.21	0.76	5.55	2.8E-08	1.5E-06
S100A9	460.67	3.01	0.59	5.08	3.7E-07	1.5E-05
SDK1	467.94	1.05	0.12	9.00	2.2E-19	5.3E-17
SECTM1	74.39	1.13	0.26	4.27	1.9E-05	4.3E-04
SEMA5A	115.07	1.15	0.22	5.24	1.6E-07	7.0E-06
SEMA6B	344.90	1.03	0.18	5.88	4.1E-09	2.6E-07
SEMA7A	132.80	-1.39	0.18	-7.71	1.2E-14	1.8E-12
SERPINB2	783.88	-2.51	0.35	-7.09	1.3E-12	1.5E-10
SERPINB7	98.29	-1.18	0.24	-4.84	1.3E-06	4.4E-05
SHANK1	53.05	-1.10	0.29	-3.77	1.7E-04	2.6E-03
SLAMF7	126.21	-1.28	0.19	-6.81	9.7E-12	9.2E-10
SLC15A3	18.77	2.20	0.59	3.72	2.0E-04	2.9E-03
SLC16A12	36.99	1.47	0.50	2.93	3.4E-03	2.7E-02
SLC28A3	20.96	-2.21	0.49	-4.53	6.0E-06	1.6E-04
SLC6A9	600.72	1.09	0.31	3.48	5.0E-04	6.1E-03
SLCO2A1	48.56	1.24	0.43	2.85	4.3E-03	3.2E-02
SLPI	221.09	1.22	0.20	6.04	1.5E-09	1.1E-07
SMAD6	23.20	2.41	0.56	4.28	1.9E-05	4.2E-04
SP140	25.33	-1.66	0.44	-3.78	1.6E-04	2.5E-03
SSC5D	88.00	2.08	0.28	7.41	1.3E-13	1.6E-11
SSPO	38.80	1.36	0.38	3.53	4.2E-04	5.3E-03
STAC2	29.84	1.49	0.43	3.46	5.4E-04	6.4E-03
STRA6	136.24	1.09	0.20	5.58	2.4E-08	1.2E-06
SULF2	201.18	1.72	0.19	9.20	3.8E-20	1.1E-17
SVEP1	448.60	1.30	0.14	9.09	9.7E-20	2.6E-17
SYT12	359.43	1.22	0.31	3.91	9.3E-05	1.6E-03
SYT17	29.93	1.43	0.42	3.45	5.6E-04	6.6E-03
TFPI2	2764.65	-1.03	0.18	-5.67	1.5E-08	8.1E-07
THSD7A	30.53	1.87	0.46	4.09	4.3E-05	8.5E-04
TJP3	83.55	1.64	0.27	6.03	1.6E-09	1.1E-07
TLX2	48.07	1.21	0.33	3.72	2.0E-04	2.9E-03
TM4SF1	1451.95	-1.20	0.08	-14.79	1.7E-49	2.9E-46
TMEM130	33.32	1.18	0.40	2.96	3.1E-03	2.5E-02
TMPRSS3	43.99	2.39	0.41	5.88	4.2E-09	2.6E-07
TRIML2	199.62	-1.09	0.38	-2.84	4.5E-03	3.3E-02
TSHZ2	97.33	1.36	0.26	5.18	2.2E-07	9.2E-06
UBD	40.70	1.07	0.34	3.11	1.9E-03	1.7E-02
UNC5C	407.59	1.47	0.13	11.18	5.0E-29	2.6E-26
VGF	391.50	-1.09	0.15	-7.09	1.3E-12	1.5E-10
VIPR2	605.32	-1.89	0.10	-18.80	8.3E-79	2.3E-75
WISP1	72.59	1.61	0.29	5.63	1.8E-08	9.6E-07
XAF1	117.73	1.40	0.23	6.05	1.5E-09	1.1E-07
ZNF323	293.84	1.07	0.14	7.57	3.9E-14	5.3E-12
ZNF467	226.47	1.50	0.17	9.11	8.5E-20	2.3E-17
ZNF521	36.98	-1.27	0.41	-3.10	2.0E-03	1.8E-02

Supplementary Table 6. GSEA (gene set enrichment analysis) in MDA-MB-436 cells using Hallmark of Cancer gene set. *P* adj, adjusted *P*. ES, enrichment score. NES, normalized enrichment score. Size represents the number of genes enriched in the dataset.

**Pathway**	**Direction**	**SIZE**	**NES**	**NOM *P***	**FDR q-val**
HALLMARK_INTERFERON_ALPHA_RESPONSE	increased in shMITF	97	1.9	0.000	0.001
HALLMARK_KRAS_SIGNALING_DN		199	1.7	0.000	0.003
HALLMARK_APICAL_SURFACE		44	1.6	0.013	0.007
HALLMARK_INTERFERON_GAMMA_RESPONSE		199	1.6	0.000	0.008
HALLMARK_BILE_ACID_METABOLISM		112	1.5	0.001	0.024
HALLMARK_ESTROGEN_RESPONSE_LATE		200	1.4	0.002	0.049
HALLMARK_MYC_TARGETS_V1	decreased in shMITF	200	-2.5	0.000	0.000
HALLMARK_MYC_TARGETS_V2		58	-2.5	0.000	0.000
HALLMARK_TNFA_SIGNALING_VIA_NFKB		200	-1.9	0.000	0.001
HALLMARK_E2F_TARGETS		200	-1.6	0.000	0.012
HALLMARK_KRAS_SIGNALING_UP		200	-1.5	0.000	0.025

Supplementary Table 7. GSEA (gene set enrichment analysis) using REACTOME gene set in MDA-MB-436 cells. *P* adj, adjusted *P*. ES, enrichment score. NES, normalized enrichment score. Size represents the number of genes enriched in the dataset.

**Pathway**		**SIZE**	**NES**	**NOM *P***	**FDR q-val**
REACTOME BETA DEFENSINS	increased in shMITF	40	1.8	0.000	0.053
REACTOME GLUCURONIDATION		18	1.8	0.000	0.040
REACTOME INTERFERON ALPHA BETASIGNALING		62	1.8	0.000	0.028
REACTOME DEFENSINS		49	1.7	0.000	0.057
REACTOME ABC FAMILY PROTEINSMEDIATED TRANSPORT		33	1.7	0.000	0.072
REACTOME COMPLEMENT CASCADE		29	1.7	0.003	0.073
REACTOME ABCA TRANSPORTERS IN LIPID HOMEOSTASIS		17	1.7	0.002	0.067
REACTOME INFLUENZA LIFE CYCLE	decreased in shMITF	136	-2.2	0.000	0.002
REACTOME ACTIVATION OF THE MRNA UPON BINDING OF THE CAP BINDING COMPLEX AND EIFS AND SUBSEQUENT BINDING TO 43S		57	-2.1	0.000	0.001
REACTOME TRANSLATION		147	-2.1	0.000	0.001
REACTOME INFLUENZA VIRAL RNA TRANSCRIPTION AND REPLICATION		102	-2.1	0.000	0.002
REACTOME 3 UTR MEDIATED TRANSLATIONAL REGULATION		106	-2.1	0.000	0.002
REACTOME PROCESSING OF CAPPED INTRON CONTAINING PRE MRNA		135	-2.1	0.000	0.002
REACTOME NONSENSE MEDIATED DECAYENHANCED BY THE EXON JUNCTION COMPLEX		107	-2.1	0.000	0.002
REACTOME FORMATION OF THE TERNARY COMPLEX AND SUBSEQUENTLY THE 43S COMPLEX		49	-2.0	0.000	0.004
REACTOME MRNA SPLICING MINOR PATHWAY		42	-2.0	0.000	0.004
REACTOME PEPTIDE CHAIN ELONGATION		86	-2.0	0.000	0.003
REACTOME MRNA PROCESSING		155	-2.0	0.000	0.003
REACTOME METABOLISM OF NONCODING RNA		48	-2.0	0.000	0.003
REACTOME METABOLISM OF RNA		256	-2.0	0.000	0.003
REACTOME MRNA SPLICING		106	-2.0	0.000	0.003
REACTOME RNA POL III TRANSCRIPTION INITIATION FROM TYPE 3 PROMOTER		26	-1.9	0.000	0.006
REACTOME FORMATION OF THE HIV1 EARLY ELONGATION COMPLEX		33	-1.9	0.000	0.008
REACTOME SRP DEPENDENT COTRANSLATIONAL PROTEIN TARGETING TO MEMBRANE		110	-1.9	0.000	0.009

Supplementary Table 8. Cell culture media.

**cell line**	**base medium**	**base medium vendor**	**base medium Cat#**	**FBS %**
RL95-2	DMEM/F-12	ATCC	30-2006	10%
Caov3	RPMI 1640	Corning Cellgro	10-040-CV	10%
OVCAR8	RPMI 1640	Corning Cellgro	10-040-CV	10%
AN3CA	EME	ATCC	30-2003	10%
Ishikawa	EME	ATCC	30-2003	10%
KLE	EME	ATCC	30-2003	10%
SNU685	DME:F12	ATCC	30-2006	15%
JHUCS-1	DME:F12	ATCC	30-2006	15%
MDA-MB-231	DMEM-HG without 2mM L glutamine	Lonza	12-614F	10%
MDA-MB-436	RPMI 1640 with 1X Glutamax	Lonza	12-614F	10%
ACI-126	DMEM/F-12	ATCC	30-2006	10%
ACI-158	DMEM/F-12	ATCC	30-2006	10%

Supplementary Table 9. The sequences of all shRNAs

shRNA (mirE)	shRNA Sequence
shRen.713	TGCTGTTGACAGTGAGCGCAGGAATTATAATGCTTATCTATAGTGAA GCCACAGATGTATAGATAAGCATTATAATTCCTATGCCTACTGCCTCGGA
shMITF.962	TGCTGTTGACAGTGAGCGAGAAGAAGAAGATTTAACATAATAGTGAAGC CACAGATGTATTATGTTAAATCTTCTTCTTCGTGCCTACTGCCTCGGA
shMITF.3309	TGCTGTTGACAGTGAGCGCTCAGATATTTTTAATATTAAATAGTGAAGCC ACAGATGTATTTAATATTAAAAATATCTGAATGCCTACTGCCTCGGA
shMITF.4514	TGCTGTTGACAGTGAGCGCGGTACTGTAATGTTAATAATATAGTGAAGCC ACAGATGTATATTATTAACATTACAGTACCATGCCTACTGCCTCGGA
shMITF.Qa	TGCTGTTGACAGTGAGCGAACCAAGTACCACATACAGCAATAGTGAAGCC ACAGATGTATTGCTGTATGTGGTACTTGGTGTGCCTACTGCCTCGGA
shMITF.Qb	TGCTGTTGACAGTGAGCGATACCACATACAGCAAGCCCAATAGTGAAGCC ACAGATGTATTGGGCTTGCTGTATGTGGTACTGCCTACTGCCTCGGA

Supplementary Table 10. qPCR primer sequences

**qPCR Primer ID**	**qPCR Primer Sequence**
MITF-FW	GAACACACATTCACGAGCG
MITF-RV	CAATCAAGTTTCCCGAGACAG
IL1A-FW	AGTGCTGCTGAAGGAGATGCCTGA
IL1A-RV	CCCCTGCCAAGCACACCCAGTA
IL1B-FW	ATGATGGCTTATTACAGTGGCAA
IL1B-RV	GTCGGAGATTCGTAGCTGGA
CD274-FW	TGGCATTTGCTGAACGCATTT
CD274-RV	TGCAGCCAGGTCTAATTGTTTT
NT5E-FW	GCCTGGGAGCTTACGATTTTG
NT5E-RV	TAGTGCCCTGGTACTGGTCG
MYC-FW	CAGCGACTCTGAGGAGGAAC
MYC-RV	GCTGTGAGGAGGTTTGCTGT
CCL2-FW	CAGCCAGATGCAATCAATGCC
CCL2-RV	TGGAATCCTGAACCCACTTCT
SVEP1-FW	GGAGCACTGTTACCTGCTACA
SVEP1-RV	TTTCCCGTAATACCCCTTTTCAC
IL15-FW	CCAGTTGCAAAGTAACAGCAA
IL15-RV	TTTTTCCTCCAGTTCCTCACA
HPRT1-FW	TGACACTGGCAAAACAATGCA
HPRT1-RV	GGTCCTTTTCACCAGCAAGCT
