# Supplementary material for: Chromatin-informed inference of transcriptional programs in gynecologic and basal breast cancers
Source: Nat Commun. 2019 Sep 25;10:4369. doi: 10.1038/s41467-019-12291-6 (PMC6761109; doi:10.1038/s41467-019-12291-6)
Supplement: Supplementary file 3 — Reporting Summary [file 41467_2019_12291_MOESM3_ESM.pdf]

nature research

Corresponding author(s): Hatice U Osmanbeyoglu, Christina S Leslie

Last updated by author(s): 2019/08/24

Reporting Summary

Nature Research wishes to improve the reproducibility of the work that we publish. This form provides structure for consistency and transparency in reporting. For further information on Nature Research policies, see Authors & Referees and the Editorial Policy Checklist.

Statistics

For all statistical analyses, confirm that the following items are present in the figure legend, table legend, main text, or Methods section.

n/a	Confirmed	
☐	☒	The exact sample size (*n*) for each experimental group/condition, given as a discrete number and unit of measurement
☐	☒	A statement on whether measurements were taken from distinct samples or whether the same sample was measured repeatedly
☐	☒	The statistical test(s) used AND whether they are one- or two-sided<br>*Only common tests should be described solely by name; describe more complex techniques in the Methods section.*
☐	☒	A description of all covariates tested
☐	☒	A description of any assumptions or corrections, such as tests of normality and adjustment for multiple comparisons
☐	☒	A full description of the statistical parameters including central tendency (e.g. means) or other basic estimates (e.g. regression coefficient) AND variation (e.g. standard deviation) or associated estimates of uncertainty (e.g. confidence intervals)
☐	☒	For null hypothesis testing, the test statistic (e.g. *F*, *t*, *r*) with confidence intervals, effect sizes, degrees of freedom and *P* value noted<br>*Give P values as exact values whenever suitable.*
☒	☐	For Bayesian analysis, information on the choice of priors and Markov chain Monte Carlo settings
☒	☐	For hierarchical and complex designs, identification of the appropriate level for tests and full reporting of outcomes
☐	☒	Estimates of effect sizes (e.g. Cohen's *d*, Pearson's *r*), indicating how they were calculated

Our web collection on statistics for biologists contains articles on many of the points above.

Software and code

Policy information about availability of computer code

Data collection	All software used for data collection are fully introduced and linked in the main-text or supplementary materials.
Data analysis	All software used for data analysis and fully introduced and linked in the main-text or supplementary materials.

For manuscripts utilizing custom algorithms or software that are central to the research but not yet described in published literature, software must be made available to editors/reviewers. We strongly encourage code deposition in a community repository (e.g. GitHub). See the Nature Research guidelines for submitting code & software for further information.

Data

Policy information about availability of data

All manuscripts must include a data availability statement. This statement should provide the following information, where applicable:

- Accession codes, unique identifiers, or web links for publicly available datasets
- A list of figures that have associated raw data
- A description of any restrictions on data availability

The authors declare that all data supporting the findings of this study are available by the links within the paper and its supplementary information files. All other relevant data is available upon request.
-The ATAC-seq and RNA-seq data have been deposited in the Gene Expression Omnibus accession number GSE114964 and GSE129337 respectively.
-RNA-seq data for each of the 5 tumor types were downloaded from TCGA's Firehose data run (https://confluence.broadinstitute.org/display/GDAC/Dashboard-Stddata).
-Cancer cell lines RNA-seq data were downloaded from the CCLE website (http://www.broadinstitute.org/ccle).
-Bigwig files of ATAC-seq profiles of tumor samples from TCGA including 13 UCEC-ENDO (24 with replicates) and 15 BRCA-BASAL (30 with replicates) were downloaded from https://gdc.cancer.gov/about-data/publications/ATACseq-AWG.
-The software for PSIONIC is available from https://sites.google.com/view/haticeulkuosmanbeyoglu/software.

Field-specific reporting

Please select the one below that is the best fit for your research. If you are not sure, read the appropriate sections before making your selection.

☒ Life sciences ☐ Behavioural & social sciences ☐ Ecological, evolutionary & environmental sciences

For a reference copy of the document with all sections, see nature.com/documents/nr-reporting-summary-flat.pdf

Life sciences study design

All studies must disclose on these points even when the disclosure is negative.

Sample size	Two replicates were used for generation ATAC-seq and RNA-seq data.
Data exclusions	No inclusion or exclusion criteria were used. No data were excluded from the study.
Replication	All results presented in the manuscript were reliably reproduced.
Randomization	Randomization is used for 10-fold cross validation analysis.
Blinding	No blinding was used.

Reporting for specific materials, systems and methods

We require information from authors about some types of materials, experimental systems and methods used in many studies. Here, indicate whether each material, system or method listed is relevant to your study. If you are not sure if a list item applies to your research, read the appropriate section before selecting a response.

Materials & experimental systems

n/a	Involved in the study	
☐	☒	Antibodies
☐	☒	Eukaryotic cell lines
☒	☐	Palaeontology
☒	☐	Animals and other organisms
☒	☐	Human research participants
☒	☐	Clinical data

Methods

n/a	Involved in the study	
☒	☐	ChIP-seq
☒	☐	Flow cytometry
☒	☐	MRI-based neuroimaging

Antibodies

Antibodies used	--anti-ETV6 antibody from Sigma (Cat# HPA000264) at the titer of 1:500;<br>--MITF (D5) clone Dako Ab. Pr Ab dilution: 1:50
Validation	All antibodies were validated by the manufacturer

Eukaryotic cell lines

Policy information about cell lines

Cell line source(s)	RL95-2(ATCC); Caov3(ATCC);OVCAR8(ATCC);AN3CA(ATCC);Ishikawa(ECACC);KLE(ATCC); SNU685(Korean Cell Line Bank);JHUCS-1(Riken);MDA-MB-231(ATCC);MDA-MB-436(ATCC);ACI-126 (John I. Risinger, Michigan State University);ACI-158(John I. Risinger, Michigan State University)
Authentication	Cells were used used for experiments after acquisition from the commercial source. ACI-126 and ACI-158 after acquisition from John I. Risinger, Michigan State University.
Mycoplasma contamination	All cell lines tested negative for mycoplasma contamination prior to use in experiments.
Commonly misidentified lines (See ICLAC register)	None of the cell lines used in this study are listed in this database.
