# Supplementary material for: Chromatin-informed inference of transcriptional programs in gynecologic and basal breast cancers
Source: Nat Commun. 2019 Sep 25;10:4369. doi: 10.1038/s41467-019-12291-6 (PMC6761109; doi:10.1038/s41467-019-12291-6)
Supplement: Supplementary file 4 — Description of Additional Supplementary Files [file 41467_2019_12291_MOESM4_ESM.pdf]

Description of Additional Supplementary Files

File Name: Supplementary Data 1
Description: Count table for the gynecologic and basal breast cancer cell lines ATACseq atlas.

File Name: Supplementary Data 2
Description: Peak motif scores.

File Name: Supplementary Data 3
Description: PSIONIC-inferred transcription factor activities of 723 TCGA ovarian, uterine, and basal breast cancer patients.

File Name: Supplementary Data 4
Description: Transcription factors that show significant tumor type specificity. We assessed TF-tumor type associations by t-test to compare inferred TF activity between samples in a given tumor type vs. those in all other tumor types. We corrected for FDR across TFs for each such pairwise comparison and identified significant TF regulators.
